# Acknowledgement to Reviewers of *International Journal of Neonatal Screening* in 2019

**DOI:** 10.3390/ijns6010003

**Published:** 2020-01-17

**Authors:** International Journal of Neonatal Screening Editorial Office

**Affiliations:** MDPI, St. Alban-Anlage 66, 4052 Basel, Switzerland

The editorial team greatly appreciates the reviewers who have dedicated their considerable time and expertise to the journal’s rigorous editorial process over the past 12 months, regardless of whether the papers are finally published or not. In 2019, a total of 35 papers were published in the journal, with a median time to first decision of 48 days and a median time from submission to publication of 18 days. The editors would like to express their sincere gratitude to the following reviewers for their generous contribution in 2019:
Aldirbashi, OsamaKingsmore, Stephen F.Anderson, SharonLeo, Carlo GiacomoAngastiniotis, MichaelLevy, HarveyArese, PaoloLobitz, StephanBonham, JimLoeber, J.GerardCaggana, MicheleLorey, FredCarew, PeterLucarelli, MarcoCarling, RachelLukacs, ZoltanCederbaum, StephenMa, Zheng FeeiCela, ElenaMartella, MaddalenaChien, Yin-HsiuMcColley, SusannaChudleigh, JaneMoat, StuartCirilli, NataliaNishio, HisahideCohen, Arieh SierraOlivieri, AntonellaCurrier, RobertOrsini, JosephDaniel, YvonnePena, LorenDankert-Roelse, Jeannette E.Ranieri, EnzoDluholucký, SvetozárRaz, AviadDocea, Anca OanaRegelmann, Molly ODucoroy, PatrickRuoppolo, MargheritaEtienne-Julan, MaryseSánchez-Pintos, PaulaFicicioglu, CanSchielen, PeterFrömmel, ClaudiaShintaku, HaruoGaviglio, AmyShone, ScottGibson, James B.Silina, YuliyaGiugliani, RobertoSpeiser, Phyllis W.Hammarström, LennartTavakoli, Norma P.Heather, NatashaTherrell, BradfordHouweling, PeterTluczek, AudreyInusa, BabaVergano, Samantha A.Jerebtsova, MarinaVon Döbeln, UlrikaKamei, DaigoWebster, DianneKavecan, IvanaWhite, Karl R.Kay, Denise M.Wiley, VeronicaKemp, StephanYamaguchi, SeijiKeutzer, JoanZetterström, RolfKharrazi, Martin


